# Nomograms for predicting long-term survival in patients with non-metastatic nasopharyngeal carcinoma in an endemic area

**DOI:** 10.18632/oncotarget.8823

**Published:** 2016-04-18

**Authors:** Qi Zeng, Ming-Huang Hong, Lu-Jun Shen, Xiang-Qi Meng, Xiang Guo, Chao-Nan Qian, Pei-Hong Wu, Pei-Yu Huang

**Affiliations:** ^1^ State Key Laboratory of Oncology in South China, Collaborative Innovation Center for Cancer Medicine, Guangzhou, PR China; ^2^ Department of Medical Imaging and Interventional Oncology, Sun Yat-sen University Cancer Center, Guangzhou, PR China; ^3^ Department of Clinical Study, Sun Yat-sen University Cancer Center, Guangzhou, PR China; ^4^ Laboratory of Tumor Microenvironment and Metastasis, Van Andel Research Institute, Grand Rapids, MI, USA; ^5^ Department of Nasopharyngeal Carcinoma, Sun Yat-sen University Cancer Center, Guangzhou, PR China

**Keywords:** nasopharyngeal carcinoma, nomogram, prognosis, radiotherapy, chemotherapy

## Abstract

**Purpose:**

Nomogram for predicting more than a 5-year survival for non-metastatic nasopharyngeal carcinoma (NPC) was lacking. This study aimed to develop the new nomograms to predict long-term survival in these patients.

**Results:**

The median follow-up time for training set and test set was 95.2 months and 133.3 months, respectively. The significant predictors for death were age, gender, body mass index (BMI), T stage, N stage, lactate dehydrogenase (LDH), and radiotherapy techniques. For predicting recurrence, age, gender, T stage, LDH, and radiotherapy techniques were significant predictors, whereas age, gender, BMI, T stage, N stage and LDH were significant predictors for distant metastasis. The calibration curves showed the good agreements between nomogram-predicted and actual survival. The c-indices for predicting death, recurrence, and distant metastases between nomograms and the TNM staging system were 0.767 VS.0.686 (P<0.001), 0.655 VS.0.585 (P<0.001), and 0.881 VS.0.754 (P<0.001), respectively. These results were further confirmed in the test set.

**Methods:**

On the basis of a retrospective study of 1593 patients (training set) who received radiotherapy alone or concurrent chemoradiotherapy from 2000 to 2004, significant predictors were identified and incorporated to build the nomograms. The calibration curves of nomogram-predicted survival versus the actual survival were plotted and reviewed. Bootstrap validation was performed to calculate the concordance index (c-index). These models were further validated in an independent prospective trial (test set, n=400).

**Conclusion:**

The established nomograms suggest more-accurate long-term prediction for patients with non-metastatic NPC.

## INTRODUCTION

Nasopharyngeal carcinoma (NPC) is a specific disease originating in the epithelial lining of the nasopharynx, with epidemiology, pathology and clinical presentation differing from other head and neck squamous cell carcinomas (HNSCC) [1]. It has a distinct geographical distribution that is closely related to the Epstein-Barr virus [2]. In endemic areas, nonkeratinizing squamous cell carcinoma accounts for 95% of cases, whereas it only accounts for 1% for keratinizing carcinoma [3]. Radiotherapy is the primary treatment for early stage disease, and concurrent chemoradiotherapy (CCRT) is the standard of care for loco-regionally advanced NPC [4].

In terms of predicting the prognosis of NPC patients, tumor-node-metastasis (TNM) classification is the most applicable staging system. However, many significant predictors, such as sex, age, lactate dehydrogenase (LDH) levels in serum, body mass index (BMI), and treatment-related factors, are not included in the TNM staging system. The nomogram is a useful predictive tool that is tailored to the profile of an individual patient and creates a more precise prediction compared to the traditional TNM staging systems [5]. In recent years, the nomogram has been used in most cancer types [6, 7]. Likewise, nomograms have been developed for NPC to generate individualized predictions [8–11]. Recently, Tang et al. developed an influential nomogram in two large samples of NPC patients [8]. However, the follow-up time in their study was less than 5 years. More importantly, a single survival endpoint that merges the death, recurrence, and distant metastases together may confuse oncologists about the failure pattern of NPC patients; whereas other similar studies have not had large enough sample sizes [10, 11] or prospective datasets [9] to validate the models. In addition, none of them provided a nomogram to predict more than a 5-year long-term survival. As a consequence, these published nomograms are not well suited for clinical practice.

The primary purpose of this study was to develop new nomograms to predict the long-term survival of patients with non-metastatic nasopharyngeal carcinoma in an endemic area and to validate the results in a prospective randomized trial.

## RESULTS

### Baseline characteristics, patterns of treatment failure, and survival

In the present study, 1593 eligible patients were analyzed in the training set, and 400 cases were included in the test set. The baseline characteristics of the patients are shown in Table 1. The final date of follow-up was April 2011 in the training set and March 2015 in the test set. Accordingly, the reverse KM estimate of the median follow-up was 95.2 months (95% CI: 93.2-97.1 months) in the training set and 133.3 months (95% CI: 130.8-135.7 months) in the test set. In the training set, 277 (17.4%) patients developed disease recurrence, 317 (19.9%) developed distant metastases, and 590 (37.0%) died. The 1-, 3-, 5- and 8-year survival rates were as follows: OS, 97.6%, 76.7%, 67.4% and 62.3%; LRFFS, 96.0%, 85.9%, 82.4% and 81.2%; and DFFS, 90.5%, 98.8%, 80.8%, 79.5% and 78.7%. In the test set, a total of 64 (16.0%) patients developed loco-regional relapse, 126 (31.5%) developed distant metastases, and 195 (48.8%) died. The 1-, 3-, 5- and 8-year survival rates were the following: OS, 97.5%, 79.9%, 71.4% and 57.4%; LRFFS, 95.9%, 89.2%, 86.3% and 80.8%; and DFFS, 90.5%, 75.1%, 70.4% and 66.9%.

**Table 1:** Baseline characteristics of patients with non-metastatic nasopharyngeal carcinoma in two datasets

Characteristics	Training set (n=1593)	Test set(n=400)
	No. of patients (%)	No. of patients (%)
**Age(y) median (range)**	46(13-78)	42(18-65)
**Gender**		
male	1210(76.0)	312(78.0)
Female	383(24.0)	88(22.0)
**BMI (kg/m2)**		
underweight (<18.5)	132(8.3)	41(10.3)
normal weight (18.5-22.9)	755(47.4)	184(46.0)
overweight (23.0-27.4)	604(37.9)	142(35.5)
obese (>27.5)	102(6.4)	33(8.3)
**Smoking status**		
never-smokers	841(52.8)	206(51.5)
ex-smokers	752(47.2)	194(48.5)
**Clinical stage**		
I	126(7.9)	0
II	646(40.6)	48(12.0)
III	580(36.4)	205(51.3)
IV	241(15.1)	147(36.8)
**T-stage**		
T1	323(20.3)	14(3.5)
T2	697(43.8)	118(29.5)
T3	369(23.2)	166(41.5)
T4	204(12.8)	102(25.5)
**N-stage**		
N0	517(32.5)	54(13.5)
N1	624(39.2)	142(35.5)
N2	409(25.7)	143(35.8)
N3	43(2.7)	61(15.3)
**Hb**		
Anemia	105(6.6)	39(9.8)
Normal	1488(93.4)	361(90.3)
**BPC**		
Thrombocytosis	233(14.6)	61(15.3)
Normal	1360(85.4)	339(84.8)
**NLR**		
<2.5	828(52.0)	-
≥2.5	765(48.0)	-
**LDH** (IU/L)		
≤245	1413(88.7)	367(91.8)
246-278	92(5.8)	12(3.0)
>278	88(5.5)	21(5.3)
**Radiotherapy techniques**		
conventional RT	1423(89.3)	400(100.0)
IMRT	170(10.7)	0
**Treatment modalities**		
RT	1066(66.9)	200(50.0)†
CCRT	527(33.1)	200(50.0)

Notes: RT: Radiotherapy alone; CCRT: concurrent chemoradiotherapy; BMI: Pre-RT weight (kg) divided by the square of height (meter); Hb: hemoglobin; BPC: blood platelet count; NLR: neutrophil-lymphocyte ratio; LDH: lactate dehydrogenase; IMRT: intensity modulated radiotherapy; †: patients received induction chemotherapy without concurrent chemotherapy.

### Independent prognostic factors in the training set

The results of the univariate analysis are listed in supplementary Table 1. Younger age, female, higher BMI, and never-smoking were associated with a better OS. Similarly, Patients who received IMRT and CCRT also had a better OS. Table 2 shows the multivariate analyses of potential predictors on OS, LRFFS, and DFFS. The results indicate age (P<0.001), gender (P<0.001), BMI group (P<0.001), T stage (P<0.001), N stage (P<0.001), LDH (P<0.001), and radiotherapy techniques (P=0.001) were independent risk factors for OS. In addition, the independent risk factors for LR-FFS were age (P<0.001), gender (P=0.013), T stage (P=0.007), LDH (P=0.001), and radiotherapy technique (P=0.001). Moreover, age (P<0.001), gender (P=0.003), BMI group (P<0.001), T stage (P=0.028), N stage (P<0.001), and LDH (P<0.001) were independent risk factors for D-FFS. However, smoking status, Hb, BPC, NLR and treatment modalities were not independent prognostic factors for survival.

**Table 2:** Multivariate analysis of the training set (n=1593)

Variable	OS			LRFFS			DFFS		
	HR	95%CI	P	HR	95%CI	P	HR	95%CI	P
**Age**									
<18	1.208	0.426-3.428	0.722	0.839	0.114-6.164	0.863	0.832	0.250-2.763	0.764
18-64	0.502	0.400-0.630	<0.001	0.506	0.365-0.703	<0.001	0.523	0.378-0.723	<0.001
≥65	Ref			Ref			Ref		
**Gender**									
male	1.472	1.187-1.824	<0.001	1.483	1.087-2.025	0.013	1.588	1.165-2.165	0.003
Female	Ref.			Ref			Ref		
**BMI**									
underweight	3.275	1.990-50392	<0.001	0.927	0.446-1.926	0.838	8.694	3.723-20.30	<0.001
normal	2.168	1.375-3.420	0.001	1.220	0.684-2.173	0.501	4.196	1.849-9.522	0.001
overweight	1.196	0.747-1.913	0.456	1.427	0.801-2.542	0.227	0.453	0.179-1.145	0.094
obese	Ref			Ref			Ref		
**Smoking status**									
never-smokers	0.855	0.724-1.011	0.067	0.841	0.636-1.112	0.224	1.166	0.899-1.512	0.248
ex-smokers	Ref			Ref			Ref		
**T-stage**									
T1	0.547	0.396-0.755	<0.001	0.537	0.341-0.846	0.007	0.594	0.373-0.946	0.028
T2	0.830	0.648-1.064	0.141	0.820	0.570-1.180	0.285	0.831	0.602-1.147	0.260
T3	0.958	0.758-1.211	0.718	0.732	0.503-1.067	0.104	1.035	0.774-1.383	0.818
T4	Ref			Ref			Ref		
**N-stage**									
N0	0.152	0.098-0.236	<0.001	0.543	0.259-1.139	0.106	0.071	0.040-0.127	<0.001
N1	0.248	0.163-0.378	<0.001	1.052	0.512-2.158	0.891	0.093	0.054-0.161	<0.001
N2	0.644	0.433-0.959	0.030	0.818	0.391-1.708	0.592	0.699	0.426-1.146	0.156
N3	Ref			Ref			Ref		
**Hb**									
Anemia	1.113	0.800-1.548	0.527	1.307	0.817-2.091	0.264	0.958	0.605-1.516	0.854
Normal	Ref			Ref			Ref		
**BPC**									
Normal	0.850	0.678-1.066	0.160	0.756	0.550-1.038	0.083	1.096	0.783-1.532	0.594
Thrombocytosis	Ref			Ref			Ref		
**NLR**									
<2.5	0.943	0.797-1.116	0.493	0.887	0.695-1.131	0.333	0.965	0.761-1.223	0.766
≥2.5	Ref			Ref			Ref		
**LDH**(IU/L)									
≤245	0.138	0.101-0.187	<0.001	0.453	0.283-0.725	0.001	0.054	0.038-0.078	<0.001
246-278	0.511	0.366-0.714	<0.001	1.001	0.561-1.785	0.997	0.163	0.108-0.247	<0.001
>278	Ref			Ref			Ref		
**RT techniques**									
conventional RT	1.684	1.221-2.322	0.001	2.583	1.471-4.536	0.001	1.382	0.927-2.059	0.112
IMRT	Ref			Ref			Ref		
**Treatment modalities**									
RT	1.187	0.983-1.433	0.076	1.198	0.901-1.591	0.213	1.166	0.911-1.494	0.223
CCRT	Ref			Ref			Ref		

Notes: RT: Radiotherapy alone; CCRT: concurrent chemoradiotherapy; BMI: Pre-RT weight (kg) divided by the square of height (meter); Hb: hemoglobin; BPC: blood platelet count; NLR: neutrophil-lymphocyte ratio; LDH: lactate dehydrogenase; IMRT: intensity modulated radiotherapy; OS: overall survival; LRFFS: locoregional failure-free survival; DFFS: distant failure-free survival; HR: hazard ratio. CI: confidence interval.

### Development and calibration of nomograms for OS, LRFFS and DFFS in the training set

**Figure 1:**
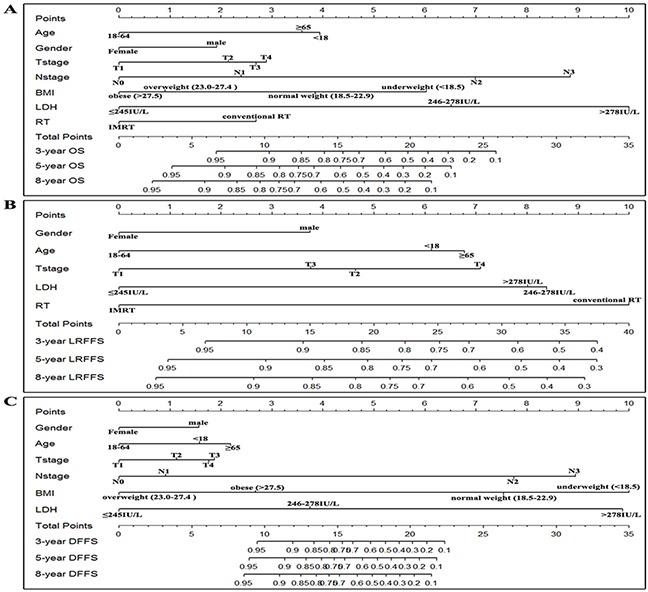
Nomograms of non-metastatic NPC patients for 3-, 5-, and 8-year OS **A.** LRFFS **B.** and DFFS **C.** BMI: body mass index; LDH: Lactate dehydrogenase; RT: radiotherapy; IMRT: intensity modulated radiotherapy; OS: overall survival; LRFFS: locoregional failure-free survival; DFFS: distant failure-free survival; Note: To make an example, locate the patient's N stage and draw a line straight upward to the “Points” axis to gain the score. Repeat the process for each predictor, and sum the scores, then locate this sum on the “Total Points” axis. Draw a line straight down to determine the probabilities of 3-, 5-, and 8-year survival.

**Figure 2:**
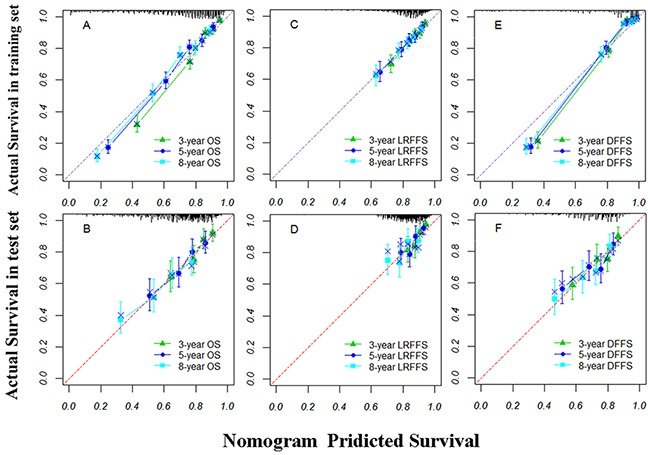
The calibration curves of nomograms for predicting overall survival (OS) at A and B locoregional failure-free survival (LRFFS) at **C** and **D.** and distant failure-free survival (DFFS) at **E** and **F.** in the training and test sets, respectively. Actual survival is plotted on the y-axis; nomogram predicted probability of survival is plotted on the x-axis. Note: A perfectly accurate nomogram prediction model would result in a plot where the observed and predicted probabilities of survival fall along the 45-degree line. the width of the CI depends on the number of patients included in each group, and it will be wider with smaller group sizes.

The prognostic nomograms that incorporated all significant independent factors for OS, LRFFS, and DFFS are presented in Figure 1A, 1B, and 1C, respectively. The nomograms illustrated that LDH and N stage have the largest contribution to OS, followed by BMI and age. For predicting the LRFFS, RT techniques had the largest impact, followed by LDH, T stage, and age. Gender also showed a moderate impact on recurrence. Additionally, BMI and LDH contributed the most to predict DFFS, with N stage following. In Figure 2, the x-axes denote the predicted probabilities of survival from the nomograms, the y-axes denote actual probabilities of survival calculated by the KM method, the 45-degree line represent the ideal reference line where the observed and predicted probabilities of survival were totally consistent. The calibration plots display optimal agreements between the nomogram-predicted survival and the actual observations for the 3-, 5-, and 8-year OS and LRFFS (Figure 2A and 2C). An excellent agreement for the 3-, 5-, and 8-year DFFS was also observed (Figure 2E).

### Comparison of predictive accuracy between nomograms and the AJCC TNM staging system

The predictive accuracy (discrimination) of a nomogram is measured by the c-index, which denotes the probability of concordance between predicted and observed responses [5]. In Table 3, internal validation using bootstrapping demonstrated the c-indices for the established nomograms to predict OS (0.767, 95% CI 0.749-0.784), LRFFS (0.655 95% CI 0.624-0.686), and DFFS (0.881, 95% CI 0.865-0.897) were significantly higher than these of the AJCC staging system (OS: 0.686, 95% CI 0.666-0.706, P<0.001; LRFFS: 0.585, 95% CI 0.554-0.617, P<0.001; DFFS: 0.754, 95% CI 0.731-0.777, P<0.001). The results indicated the nomogram model significantly improved predictive accuracy compared with the AJCC staging system.

**Table 3:** Comparison of C-indices in the training set and test set

	Training set(n=1593)		P	Test set(n=400)		P
	Nomogram model	AJCC staging system		Nomogram model	AJCC staging system	
	c-index(95%CI)	c-index(95%CI)		c-index(95%CI)	c-index(95%CI)	
OS	0.767(0.749-0.784)	0.686(0.666-0.706)	P<0.001	0.657(0.618-0.695)	0.602(0.561-0.643)	P<0.001
LRFFS	0.655(0.624-0.686)	0.585(0.554-0.617)	P<0.001	0.643(0.581-0.705)	0.598(0.531-0.666)	P=0.001
DFFS	0.881(0.865-0.897)	0.754(0.731-0.777)	P<0.001	0.635(0.587-0.684)	0.591(0.542-0.641)	P<0.001

Notes: OS: overall survival; LRFFS: locoregional failure-free survival; DFFS: distant failure-free survival; AJCC: American

Joint Committee on Cancer; c-index: Harrell's concordance index

### Validation of predictive accuracy of the nomograms for OS, LRFFS and DFFS

In the test set, the C-indices were also significantly improved for the nomogram prediction compared with the AJCC staging system (OS: [0.657, 95% CI 0.618-0.695] VS. [0.602, 95% CI 0.561-0.643], P<0.001; LRFFS: [0.643, 95% CI 0.581-0.705] VS. [0.598, 95% CI 0.531-0.666], P=0.001; DFFS: [0.635, 95% CI 0.587-0.684] VS. [0.591, 95% CI 0.542-0.641], P=0.001). The calibration plots show the optimal agreement of the probability of survival between the nomogram prediction and the actual observation for the 3-, 5-, and 8-year OS, LRFFS, and DFFS (Figure 2B, 2D, and 2F). All these results demonstrate that the established nomograms in the training set can be well validated in the test set.

### Risk stratification of death, recurrence, and distant metastasis for patients with non-metastatic nasopharyngeal carcinoma

**Figure 3:**
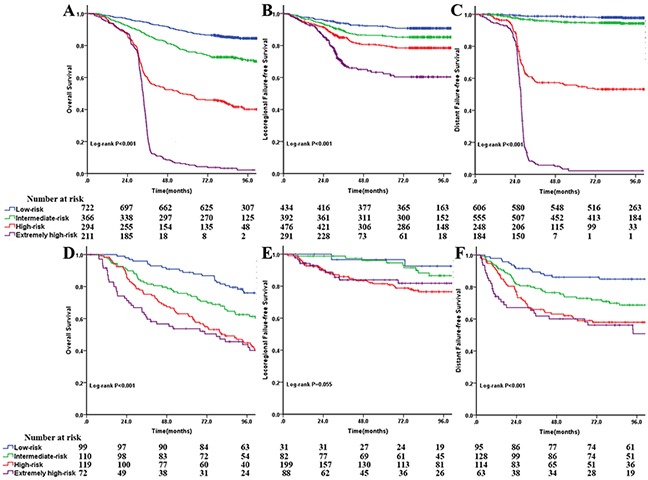
Comparison of Kaplan-Meier survival curves among four risk groups for overall survival at A and D locoregional failure-free survival at **B** and **E.** and distant failure-free survival (C and F) in the training and test sets, respectively.

Based on the cutoff values of the total points determined by ROC curve, patients were classified into a low-risk group (<13.5), intermediate-risk group (13.5-16.5), high-risk group (16.5-20.5), and extremely high-risk group (≥20.5) for death. For predicting loco-regional recurrence, the cutoff values were 12.5, 14.5, and 17.5, respectively. The points for low-risk, intermediate-risk, high-risk, and extremely high-risk distant metastasis were <9.5, 9.5-13.5, 13.5-17.5, and ≥17.5, respectively. As illustrated in Figure 3, the 8-year OS among the four risk groups were 87.7%, 78.9%, 59.5%, and 13.5% (P<0.001, Figure 3A), respectively. Likewise, there were significant differences for LRFFS (P<0.001, Figure 3B) and DFFS (P<0.001, Figure 3C). Our results showed this risk stratification in the training set could effectively discriminate the survival.

In the test set, the risk stratification of death and distant metastasis remains a useful instrument to separate the patients with different survival, although the differences between the high-risk group and the extremely high-risk group were not significant (Figure 3D and 3F). Similarly, the differences were not significant for LRFFS between the low-risk and intermediate-risk groups and the high-risk and extremely high-risk groups (Figure 3E), largely due to the fairly small sample size in the cohort.

## DISCUSSION

The treatment results of NPC were relatively good, owing to its high sensitive to radiotherapy and chemotherapy [3]. Previous studies showed approximately 49.5% to 66% of patients would survive at least 10 years [12, 13]. Since the introduction of IMRT combined with concurrent chemotherapy, the survival and quality of life have been further improved with less late toxicity [4, 14, 15]. Thus the new nomograms that predict more than a 5-year long-term survival of patients with or without concurrent chemotherapy are warranted in order to guide individualized treatments. In the present study, the median follow-up was 95.2 months in the training set and 133.3 months in the test set, which guaranteed that we identified the significant prognostic factors for 8-year survival. Next, the maximum 8-year survival prediction model was developed, which was superior to the TNM staging system (Table 3). More importantly, a prospective trial was used to validate the established nomograms, confirming their predictive accuracy (Figure 2).

To review the published nomograms for predicting 5-year NPC survival in large sample studies [8, 9], the N stage contributed the most to predict death and distant metastasis. In our study, the N stage also had the leading power in predicting the 8-year OS and DFFS. In addition, there has been some controversy about the role of Epstein-barr virus DNA (EBV-DNA). Tang and colleagues have indicated that the addition of EBV-DNA into the model contributed the most, even over the N stage [8]. However, another study showed that the EBV-DNA only contributes a little, with a maximum of 22 points compared to the 100 points of N3 [9]. In our study, we did not include the EBV-DNA because our data were from 2000 to 2005. At that time, measuring EBV-DNA was not common in the majority of institutions. Although EBV-DNA has been considered to be a useful biomarker for NPC patients [16, 17], no other evidence has shown EBV-DNA to outperform N stage as a predictor. Even in Tang's study, the c-indices of the model with or without EBV-DNA in the validation set are not significantly different (P=0.09). In our study, the c-indices (Table 3) for OS and DFFS were larger than those of the previous studies [8, 9]. Our results were further validated in the prospective trial.

LDH and BMI were also significant predictors for NPC, which have been confirmed in previous prospective and retrospective data [10, 18, 19]. Our results showed that LDH contributed greatly to predict OS, LRFFSand DFFS, while BMI contributed the most for predicting the DFFS. These findings were similar to a previous study [8]. It is plausible that patients with a higher BMI could withstand aggressive combined chemo-radiotherapy and have relatively good survival [18]. It is noteworthy that previous studies [10, 19] have suggested that elevated LDH levels may reflect a large tumor burden, which may interact with T stage. Thus, the covariate (LDH*T stage) was included in the multivariate analysis. No interaction between LDH and T stage was found (data not shown). Altogether, N stage combined with other biomarkers and individual parameters has provided a good model to predict the OS and DFFS.

With the advance of radiotherapy techniques, IMRT has been recommended by the NCCN guideline for NPC patients in clinical practice. However, IMRT is still not routinely available in economically challenged regions. Thus, this study incorporated the radiotherapy techniques (conventional RT VS. IMRT) in the model, which will increase its scope of application. As shown in Figure 1B, IMRT and LDH made the greatest contribution to predicting loco-regional recurrence. These findings are consistent with previous studies [12, 14, 19] that demonstrated that IMRT could obviously improve loco-regional control compared to conventional RT.

A recent meta-analysis [4] that comprised 19 trials and 4806 patients confirmed that CCRT plus AC and CCRT without AC significantly improved OS compared with AC alone or IC alone. Thus, we just selected patients who received a CCRT-based regimen. The patients receiving IC and/or AC alone were excluded. Remarkably, none of the published nomograms incorporated the CCRT into the model because CCRT was not a significant predictor in these studies [8–11]. We had similar findings in the present study. A possible explanation for this was primarily that the training data sets in all these studies were retrospective, which made it difficult to find positive results. Additionally, the sample sizes were still not enough to reveal the differences.

We further categorized patients into four risk groups in two data sets. In general, the survival curves separated very well except for LRFFS in the test set (P=0.055). The relatively small sample size in the cohort may contribute to its insignificance. Despite this, the results showed that a higher number of total points was associated with a higher risk of death, loco-regional recurrence, and distant metastases. Consequently, patients in high-risk and extremely high-risk groups should receive special attention and active surveillance.

Our study had several limitations. First, some molecular markers, such as vascular endothelial growth factor (VEGF) and epidermal growth factor receptor (EGFR), which can be found in over 60% of NPC patients, were not included in the nomograms [20, 21]. Both of them were associated with the survival of NPC patients [22, 23]. However, the data were unattainable, given the expense of genetic testing. Second, all the data were from only one cancer center in the endemic area. Thus, special attention should be paid to apply the nomograms to patients with keratinizing carcinoma in non-endemic regions. Finally, similar to published nomograms, our study did not incorporate the concurrent chemotherapy into the model, which compromised the real application in clinical practice. Further studies are needed to elaborate on the addition of CCRT to nomograms.

To our knowledge, this is the first study to develop nomograms for predicting 8-year OS, LRFFS, and DFFS in patients with non-metastatic nasopharyngeal carcinoma. Compared to published nomograms, more survival endpoints in our study will further promote the understanding of patterns of treatment failure for NPC. In addition, significant predictors of death, loco-regional and distant recurrence were confirmed, which will provide an important reference for future clinical trials.

In summary, the present clinical study, with the median follow-up time of 95.2 months in the training set and 133.3 months in the test set, found that the significant predictors for death were age, gender, BMI group, T stage, N stage, LDH, and radiotherapy technique. For predicting loco-regional recurrence, age, gender, T stage, LDH, and radiotherapy techniques were significant predictors. Afterwards, age, gender, BMI group, T stage, N stage and LDH were found to be significant predictors of distant metastasis. Then, the new nomograms were established and validated with optimal agreement between nomogram-predicted and actual observed survival, which further demonstrates the superior discrimination compared to the AJCC TNM staging system. In addition, our nomograms suggest that patients in high-risk and extremely high-risk groups should receive more attention given the higher death rate and the recurrence and distant metastases. It is hoped that the current nomograms can be further applied and validated in the clinical practices of other institutions.

## MATERIALS AND METHODS

### Patient and data processing

A retrospective study (training set) consisting of 1593 patients who underwent treatment in our cancer center from November 2000 to December 2004 was established. The inclusion criteria were as follows: (i) histologically confirmed nonkeratinizing; (ii) patients without systemic metastasis at the time of diagnosis; (iii) patients who received definitive RT alone or combined with chemoradiotherapy (concurrent chemotherapy-based regimen). Patients with incomplete documents and follow-up, second primary malignancy, and serious comorbidities (grade 2-3) were excluded. The study was approved by the review board of our institute and the Hospital Ethics Committee. The ethics committee granted a waiver of individual informed consent because this was an analysis of routine data. All patients were evaluated through the pretreatment examinations: physical examination, fiberoptic endoscope examination of the nasopharynx, complete blood count and biochemistry test, CT or MRI of the nasopharynx and neck, chest radiography, abdominal ultrasound, and bone scintigraphy. Patients were restaged according to the sixth edition of the American Joint Committee on Cancer (AJCC) staging system.

Based on clinical findings in previous large samples or prospective studies [10, 18, 19], potential predictors were collected that included age, gender, BMI (pretreatment weight divided by the square of the height), smoking status at diagnosis, hemoglobin (Hb), blood platelet count (BPC), neutrophil-lymphocyte ratio (NLR), LDH, RT techniques, and treatment modalities. Continuous variables were transformed into categorical variables based on previous studies. Age was separated into three groups with <18, 18-64, ≥65. The BMI was divided into four groups according to the World Health Organization classifications for the Asian populations [24]. The cutoff point for NLR was 2.5 [25]. Anemia was defined as Hb≤120 g/L (women) or Hb≤130 g/L (men). Thrombocytosis was defined as BPC>300*10^9^/L. LDH was categorized into 3 groups according to the upper limit of normal (245IU/L) and the median of patients with LDH>245IU/L. Smoking status was split into never-smokers and ex-smokers [12].

Between August 2002 and April 2005, an independent cohort (test set) composed of 400 patients with loco-regionally advanced nasopharyngeal carcinoma was prospectively studied. Details of this randomized trial, including the inclusion/exclusion criteria, randomization, statistical methods, and results, have been reported previously [26]. It should be noted that all the predictors in the trial were collected prospectively. The trial was used to test the validity of the nomogram.

### Treatment

During the study, the RT technique in our cancer center evolved from a conventional RT to intensity modulated radiotherapy (IMRT). These details of the RT technique have been previously described [14, 27, 28]. In summary, conventional radiation therapy (n=1423) was performed with a cumulative dose of over 66Gy to the gross tumor (2 Gy each fraction with five fractions per week), 60 to 66 Gy to the involved areas of the neck and over 50 Gy to the uninvolved areas. When using IMRT (n=170), 68Gy was prescribed to the gross tumor volume of the nasopharynx (GTVnx) and 60 to 64 Gy to the metastatic lymph nodes (GTVnd). In a test set, all the patients received conventional radiation therapy. Combined modality therapy in training set for loco-regionally advanced NPC included CCRT (n=306), induction chemotherapy (IC) +CCRT (n=184), IC+CCRT+ adjuvant chemotherapy (AC, n=14), and CCRT+AC (n=23). The concurrent chemotherapy regimen was cisplatin alone, with cisplatin (80-100 mg/m^2^) given intravenously 3 times weekly for three cycles or cisplatin (30-40 mg/m^2^ on day 1) given intravenously weekly for 5-7 cycles. Patients in a test set received IC+CCRT (n=201) or IC+RT (n=199), respectively. The IC regimen was floxuridine (FuDR) + carboplatin (CBP) (FuDR, 750 mg/m2, d1-5; CBP, area under the curve [AUC] = 6) for two cycles; whereas the concurrent chemotherapy was carboplatin (AUC = 6) for three cycles.

### Follow-up and end points

After treatment, patients were observed once every three months in the first three years, every six months until the fifth year thereafter, and then the follow-up intervals increased to 12 to 24 months after 5 years. At the time of each follow-up visit, a complete physical examination and imaging tests were performed. The site and timing of tumor relapse and/or metastasis were documented. The follow-up duration in the training set was calculated from the day of therapy start and from a randomization of the test set. Overall survival (OS) was used as the primary end point. OS was defined as the time until death from any cause or the last date of follow-up. Additionally, we also observed secondary endpoints including loco-regional failure-free survival (LRFFS) as well as distant failure-free survival (DFFS). LRFFS and DFFS were defined as the time to loco-regional or remote failure, respectively.

### Statistical analysis

Descriptive statistics were performed to define the baseline characteristics of patients in the two data sets. A method of the reverse Kaplan-Meier (KM) estimator was used to calculate the median follow-up time [29]. Receiver operating characteristic (ROC) analysis based on a weighted Youden index was used to determine the cutoff values for risk stratification of survival. Survival curves were described by the KM method and compared using a log-rank test. All the candidate predictors were included in the univariable and multivariable analyses with a Cox proportional hazards model. The independent prognostic factors for OS, LRFFS, and DFFS in the training set were obtained with the stepwise backward elimination. All the statistical analyses were performed using the SPSS statistical software program version 19.0 (SPSS Inc., an IBM Company; Chicago, IL, USA). Furthermore, the nomograms to predict death, loco-regional recurrence, and distant metastasis were generated by using the rms package in R version 3.1.3 (R Foundation for Statistical Computing, Vienna, Austria). The final model was obtained with a backward stepwise selection procedure using Akaike Information Criterion (AIC) [5]. The validity of these nomograms was assessed by calibration and discrimination. The calibration curve was developed to compare nomogram-predicted survival with Kaplan-Meier estimates of survival. In addition, Harrell's concordance index (c-index) was calculated by the Hmisc package (rcorrp.cens) in R (www.r-project.org) and compared to discriminate the established nomogram model from the TNM staging system. The bootstrapping technique was used to internally validate the model. The established nomograms were further validated in a prospective clinical trial (the test set). Finally, the total points of each patient were calculated based on the established nomograms and then categorized into four risk groups. The survival of the four groups was compared in the training and test sets. All tests were considered to be statistically significant at a level of 0.05. The related R language programs for nomogram calculation are listed in the Appendix (online only).

## SUPPLEMENTARY MATERIALS TABLES



## References

[1] Young LS, Dawson CW (2014). Epstein-Barr virus and nasopharyngeal carcinoma. Chin J Cancer.

[2] Wei KR, Zheng RS, Zhang SW, Liang ZH, Ou ZX, Chen WQ (2014). Nasopharyngeal carcinoma incidence and mortality in China in 2010. Chin J Cancer.

[3] Wei WI, Sham JS (2005). Nasopharyngeal carcinoma. LANCET.

[4] Blanchard P, Lee A, Marguet S, Leclercq J, Ng WT, Ma J, Chan AT, Huang PY, Benhamou E, Zhu G, Chua DT, Chen Y, Mai HQ, Kwong DL, Cheah SL, Moon J (2015). Chemotherapy and radiotherapy in nasopharyngeal carcinoma: an update of the MAC-NPC meta-analysis. LANCET ONCOL.

[5] Iasonos A, Schrag D, Raj GV, Panageas KS (2008). How to build and interpret a nomogram for cancer prognosis. J CLIN ONCOL.

[6] Liang W, Zhang L, Jiang G, Wang Q, Liu L, Liu D, Wang Z, Zhu Z, Deng Q, Xiong X, Shao W, Shi X, He J (2015). Development and validation of a nomogram for predicting survival in patients with resected non-small-cell lung cancer. J CLIN ONCOL.

[7] Wang Y, Li J, Xia Y, Gong R, Wang K, Yan Z, Wan X, Liu G, Wu D, Shi L, Lau W, Wu M, Shen F (2013). Prognostic nomogram for intrahepatic cholangiocarcinoma after partial hepatectomy. J CLIN ONCOL.

[8] Tang LQ, Li CF, Li J, Chen WH, Chen QY, Yuan LX, Lai XP, He Y, Xu YX, Hu DP, Wen SH, Peng YT, Zhang L, Guo SS, Liu LT, Guo L (2016). Establishment and Validation of Prognostic Nomograms for Endemic Nasopharyngeal Carcinoma. J NATL CANCER INST.

[9] Yang L, Hong S, Wang Y, Chen H, Liang S, Peng P, Chen Y (2015). Development and External Validation of Nomograms for Predicting Survival in Nasopharyngeal Carcinoma Patients after Definitive Radiotherapy. SCI REP.

[10] Zeng L, Guo P, Li JG, Han F, Li Q, Lu Y, Deng XW, Zhang QY, Lu TX (2015). Prognostic score models for survival of nasopharyngeal carcinoma patients treated with intensity-modulated radiotherapy and chemotherapy. ONCOTARGET.

[11] Cho JK, Lee GJ, Yi KI, Cho KS, Choi N, Kim JS, Kim H, Oh D, Choi SK, Jung SH, Jeong HS, Ahn YC (2015). Development and external validation of nomograms predictive of response to radiation therapy and overall survival in nasopharyngeal cancer patients. EUR J CANCER.

[12] Huang PY, Zeng Q, Cao KJ, Guo X, Guo L, Mo HY, Wu PH, Qian CN, Mai HQ, Hong MH (2015). Ten-year outcomes of a randomised trial for locoregionally advanced nasopharyngeal carcinoma: A single-institution experience from an endemic area. EUR J CANCER.

[13] Yi JL, Gao L, Huang XD, Li SY, Luo JW, Cai WM, Xiao JP, Xu GZ (2006). Nasopharyngeal carcinoma treated by radical radiotherapy alone: Ten-year experience of a single institution. INT J RADIAT ONCOL BIOL PHYS.

[14] Zhang MX, Li J, Shen GP, Zou X, Xu JJ, Jiang R, You R, Hua YJ, Sun Y, Ma J, Hong MH, Chen MY (2015). Intensity-modulated radiotherapy prolongs the survival of patients with nasopharyngeal carcinoma compared with conventional two-dimensional radiotherapy: A 10-year experience with a large cohort and long follow-up. EUR J CANCER.

[15] Kwong DL, Pow EH, Sham JS, McMillan AS, Leung LH, Leung WK, Chua DT, Cheng AC, Wu PM, Au GK (2004). Intensity-modulated radiotherapy for early-stage nasopharyngeal carcinoma: a prospective study on disease control and preservation of salivary function. CANCER-AM CANCER SOC.

[16] Song C, Yang S (2013). A meta-analysis on the EBV DNA and VCA-IgA in diagnosis of Nasopharyngeal Carcinoma. PAK J MED SCI.

[17] Leung SF, Zee B, Ma BB, Hui EP, Mo F, Lai M, Chan KC, Chan LY, Kwan WH, Lo YM, Chan AT (2006). Plasma Epstein-Barr viral deoxyribonucleic acid quantitation complements tumor-node-metastasis staging prognostication in nasopharyngeal carcinoma. J CLIN ONCOL.

[18] Huang PY, Wang CT, Cao KJ, Guo X, Guo L, Mo HY, Wen BX, Wu YS, Mai HQ, Hong MH (2013). Pretreatment body mass index as an independent prognostic factor in patients with locoregionally advanced nasopharyngeal carcinoma treated with chemoradiotherapy: findings from a randomised trial. EUR J CANCER.

[19] Wan XB, Wei L, Li H, Dong M, Lin Q, Ma XK, Huang PY, Wen JY, Li X, Chen J, Ruan DY, Lin ZX, Chen ZH, Liu Q, Wu XY, Hong MH (2013). High pretreatment serum lactate dehydrogenase level correlates with disease relapse and predicts an inferior outcome in locally advanced nasopharyngeal carcinoma. EUR J CANCER.

[20] Ma BB, Kam MK, Leung SF, Hui EP, King AD, Chan SL, Mo F, Loong H, Yu BK, Ahuja A, Chan AT (2012). A phase II study of concurrent cetuximab-cisplatin and intensity-modulated radiotherapy in locoregionally advanced nasopharyngeal carcinoma. ANN ONCOL.

[21] Krishna SM, James S, Balaram P (2006). Expression of VEGF as prognosticator in primary nasopharyngeal cancer and its relation to EBV status. VIRUS RES.

[22] Kyzas PA, Cunha IW, Ioannidis JP (2005). Prognostic significance of vascular endothelial growth factor immunohistochemical expression in head and neck squamous cell carcinoma: a meta-analysis. CLIN CANCER RES.

[23] Ma BB, Poon TC, To KF, Zee B, Mo FK, Chan CM, Ho S, Teo PM, Johnson PJ, Chan AT (2003). Prognostic significance of tumor angiogenesis, Ki 67, p53 oncoprotein, epidermal growth factor receptor and HER2 receptor protein expression in undifferentiated nasopharyngeal carcinoma--a prospective study. HEAD NECK.

[24] Appropriate body-mass index for Asian populations its implications for policy and intervention strategies (2004). LANCET.

[25] Chang H, Gao J, Xu BQ, Guo SP, Lu RB, Li G, Huang SM, Han F, Liu ZG, Tao YL, Tu ZW, Chen C, Li XH, Xia YF (2013). Haemoglobin, neutrophil to lymphocyte ratio and platelet count improve prognosis prediction of the TNM staging system in nasopharyngeal carcinoma: development and validation in 3,237 patients from a single institution. CLIN ONCOL (R COLL RADIOL).

[26] Huang PY, Cao KJ, Guo X, Mo HY, Guo L, Xiang YQ, Deng MQ, Qiu F, Cao SM, Guo Y, Zhang L, Li NW, Sun R, Chen QY, Luo DH, Hua YJ (2012). A randomized trial of induction chemotherapy plus concurrent chemoradiotherapy versus induction chemotherapy plus radiotherapy for locoregionally advanced nasopharyngeal carcinoma. ORAL ONCOL.

[27] Zheng Y, Han F, Xiao W, Xiang Y, Lu L, Deng X, Cui N, Zhao C (2015). Analysis of late toxicity in nasopharyngeal carcinoma patients treated with intensity modulated radiation therapy. RADIAT ONCOL.

[28] Chen L, Hu CS, Chen XZ, Hu GQ, Cheng ZB, Sun Y, Li WX, Chen YY, Xie FY, Liang SB, Chen Y, Xu TT, Li B, Long GX, Wang SY, Zheng BM (2012). Concurrent chemoradiotherapy plus adjuvant chemotherapy versus concurrent chemoradiotherapy alone in patients with locoregionally advanced nasopharyngeal carcinoma: a phase 3 multicentre randomised controlled trial. LANCET ONCOL.

[29] Schemper M, Smith TL (1996). A note on quantifying follow-up in studies of failure time. CONTROL CLIN TRIALS.

