# Supplementary material for: Nomograms for predicting long-term survival in patients with non-metastatic nasopharyngeal carcinoma in an endemic area
Source: Oncotarget. 2016 Apr 18;7(20):29708–19. doi: 10.18632/oncotarget.8823 (PMC5045427; doi:10.18632/oncotarget.8823)
Supplement: Supplementary file 1 [file oncotarget-07-29708-s001.pdf]

SUPPLEMENTARY TABLES

Supplementary Table S1: Univariate analyses in the training set

Variable	OS		LRFFS		DFFS	
	HR(95%CI)	P	HR(95%CI)	P	HR 95%CI	P
**Age**						
<18	1.961(0.720-5.339)	0.187	0.790(0.109-5.732)	0.816	3.248(1.008-10.46)	0.048
18-64	0.431(0.345-0.538)	<0.001	0.434(0.315-0.597)	<0.001	0.494(0.361-0.678)	<0.001
≥65	Ref		Ref		Ref	
**Gender**						
male	1.503(1.222-1.849)	<0.001	1.499(1.106-2.032)	0.009	1.620(1.212-2.167)	0.001
Female	Ref		Ref		Ref	
**BMI**						
underweight	5.206(3.203-8.460)	<0.001	1.423(0.702-2.883)	0.328	14.35(6.248-32.97)	<0.001
normal	2.749(1.750-4.316)	<0.001	1.509(0.854-2.669)	0.157	5.778(2.567-13.01)	<0.001
overweight	1.277(0.800-2.039)	0.305	1.507(0.849-2.675)	0.161	0.520(0.206-1.309)	0.165
obese	Ref		Ref		Ref	
**Smoking status**						
never-smokers	0.756(0.643-0.889)	0.001	0.693(0.547-0.877)	0.002	0.784(0.629-0.977)	0.030
ex-smokers	Ref		Ref		Ref	
**T-stage**						
T1	0.236(0.175-0.319)	<0.001	0.373(0.245-0.569)	<0.001	0.148(0.096-0.230)	<0.001
T2	0.405(0.326-0.505)	<0.001	0.606(0.437-0.840)	0.003	0.263(0.196-0.353)	<0.001
T3	0.686(0.545-0.863)	0.001	0.617(0.426-0.893)	0.010	0.657(0.496-0.870)	0.003
T4	Ref		Ref		Ref	
**N-stage**						
N0	0.215(0.143-0.322)	<0.001	0.518(0.249-1.080)	0.079	0.179(0.105-0.304)	<0.001
N1	0.344(0.233-0.507)	<0.001	0.968(0.474-1.975)	0.929	0.231(0.139-0.385)	<0.001
N2	0.706(0.480-1.039)	0.078	0.765(0.367-1.596)	0.476	0.834(0.512-1.358)	0.465
N3	Ref		Ref		Ref	
**Hb**						
Anemia	1.146(0.841-1.564)	0.388	1.197(0.767-1.867)	0.429	1.188(0.784-1.802)	0.416
Normal	Ref		Ref		Ref	
**BPC**						
Normal	0.886(0.710-1.104)	0.281	0.789(0.578-1.077)	0.135	1.064(0.771-1.467)	0.707
Thrombocytosis	Ref		Ref		Ref	
**NLR**						
<2.5	0.691(0.588-0.813)	<0.001	0.771(0.609-0.976)	0.031	0.593(0.474-0.741)	<0.001
≥2.5	Ref		Ref		Ref	

(*Continued*)

**Variable**	**OS**		**LRFFS**		**DFFS**	
	**HR(95%CI)**	**P**	**HR(95%CI)**	**P**	**HR 95%CI**	**P**
**LDH(IU/L)**						
≤245	0.117(0.091-0.149)	<0.001	0.354(0.234-0.536)	<0.001	0.053(0.040-0.070)	<0.001
246-278	0.784(0.584-1.053)	0.105	0.864(0.499-1.498)	0.604	0.493(0.356-0.684)	<0.001
>278	Ref		Ref		Ref	
**RT techniques**						
conventional RT	1.678(1.226-2.297)	0.001	2.678(1.535-4.674)	0.001	1.403(0.946-2.082)	0.092
IMRT	Ref		Ref		Ref	
**Treatment modalities**						
RT	0.642(0.545-0.757)	<0.001	1.002(0.778-1.291)	0.987	0.493(0.395-0.614)	<0.001
CCRT	Ref		Ref		Ref	

Notes: RT: Radiotherapy alone; CCRT: concurrent chemoradiotherapy; BMI: Pre-RT weight (kg) divided by the square of height (meter); Hb: hemoglobin; BPC: blood platelet count; NLR: neutrop hil-lymphocyte ratio; LDH: lactate dehydrogenase; IMRT: intensity modulated radiotherapy; OS: overall survival; LRFFS: locoregional failure-free survival; DFFS: distant failure-free survival; HR: hazard ratio. CI: confidence interval.
